# Diurnal Preference and Grey Matter Volume in a Large Population of Older Adults: Data from the UK Biobank

**DOI:** 10.5334/jcr.193

**Published:** 2020-05-08

**Authors:** Ray Norbury

**Affiliations:** 1 College of Health and Life Sciences, Division of Psychology, Brunel University London, Uxbridge, UK

**Keywords:** Eveningness, chronotype, anatomy, VBM, Biobank

## Abstract

Eveningness (a diurnal preference for evening time) is associated with a number of negative health outcomes and risk and prevalence for psychiatric disorder. Our understanding of the anatomical substrates of diurnal preference, however, is limited. The current study used Voxel-Based Morphometry to compare grey matter volume in a large sample (*N* = 3730) of healthy adults determined by questionnaire to be either definite morning-type or definite evening-type. Eveningness was associated with increased grey matter volume in precuneus, brain regions implicated in risk and reward processing (bilateral nucleus accumbens, caudate, putamen and thalamus) and orbitofrontal cortex. These results indicate an anatomical-basis for diurnal preference which may underlie reported differences in behaviour and brain function observed in these individuals.

## Introduction

Circadian rhythms are near-24-hour oscillations that have been observed throughout the brain and body [1]. Set by the master pacemaker in the suprachiasmatic nucleus (SCN) the circadian timing system synchronises peripheral clocks throughout the body to adapt and optimise physiological processes in response to changes in the environment [2].

Circadian behavioural phenotypes can be determined using self-report measures of diurnal preference (i.e. a tendency towards more morningness or eveningness [3]) or chronotype (morning- vs. evening-type sleep schedules [4]). Using these instruments, a broadening corpus suggests that eveningness (late chronotype) is associated with a number of negative health outcomes (e.g. type-2 diabetes and cardiovascular disease [5]), increased risk for psychiatric disorder [6,7] and personality-traits such as neuroticism [8]. By contrast, morningness (early chronotype) is associated with increased conscientiousness, openness and agreeability [8] and reduced procrastination [9].

The functional and structural substrates of diurnal preference and chronotype are poorly understood, largely due to the paucity of studies exploring these questions. Functional Magnetic Resonance Imaging (FMRI) studies of circadian typology have reported effects of chronotype/diurnal preference on attention [10,11], language comprehension [12], inhibition [13], working memory [14], reward processing [15,16], emotional processing [17] and resting connectivity [18,19] – although see Fafrowicz [20] who reported no effect of diurnal preference on resting-state connectivity as measured by ALFF (Amplitude of Low Frequency Fluctuations – an index of total power within a predefined frequency range [20]). Interpretation of the above findings is limited due to the relatively small number of studies and, to date, lack of replication. Nevertheless, these data complement previous behavioural work demonstrating effects of diurnal preference on cognition, emotional processing [21,22,23], reward processing and risk-taking [24,25] and epidemiological studies reporting eveningness/late chronotype as a risk factor for a number of psychiatric disorders [7,26].

To date only three studies have used whole-brain, voxel-wise measures to examine the structural determinants of circadian typology. Rosenberg and colleagues investigated the impact of chronotype on anatomical connectivity using Diffusion Tensor Imaging (DTI) in a population of 59 18–35 year old males [27]. Compared to early chronotypes, late types had reduced microstructural integrity (lower fractional anisotropy values) in white matter underlying the cingulate gyrus and frontal lobe and greater mean diffusivity (which may reflect localised reductions in neuropil) in frontal regions and precentral gyrus. Takeuchi *et al*., using voxel-based morphometry (VBM) in a large sample (N = 776) of young adults (mean age 21), reported a negative correlation between Morningness-Eveningness (MEQ) score and grey matter volume in precuneus and posterior parietal cortex and a positive association in bilateral orbitofrontal cortex [28]. The authors also reported a positive correlation between MEQ and grey matter volume in bilateral suprachiasmatic nuclei although these latter results were uncorrected for multiple comparisons. More recently, Rosenberg *et al*., replicated the precuneus finding in a sample of 48 male participants aged 18–35 years. The authors further reported greater grey matter volume in lateral occipital cortex in late chronotypes as compared to early types and greater cortical thickness in left anterior insula, inferior parietal cortex, and right pars triangularis [29].

The goal of the current study was to explore the structural determinants of diurnal preference in a large sample of older adults using VBM. Based on the extant, although limited, literature it was hypothesised that eveningness would be associated with greater grey-matter volume in precuneus. An additional whole-brain *post hoc* analysis was also conducted and will be treated as hypothesis-generating (exploratory).

## Methods

### Participants

Data were taken from the UK Biobank Resource. Ethical approval to the UK Biobank was granted by the NHS National Research Ethics Service North West (Reference number: 11/NW/0382). The current study was approved by the UK Biobank Access Committee (Project reference number 30833).

### Measures

The principal exposure variable was diurnal preference which was assessed in the Biobank cohort with the single question: “Do you consider yourself to be definitely a morning person/more a morning than an evening person/more an evening than a morning person/definitely an evening person”. The current study included participants who self-reported as “Definitely an ‘evening’ person” or “Definitely a ‘morning’ person”. Participants who answered “Do not know” or “Prefer not to answer” were also excluded. Age, sex and sleep duration (determined using the question “About how many hours sleep do you get in every 24 hours, please include naps?” with values provided as integers) were also recorded.

Mental health was assessed with the following question: “Have you been diagnosed with one or more of the following mental health problems by a professional, even if you don’t have it currently? Participants were also provided with following clarification statement: “By professional we mean: any doctor, nurse or person with specialist training (such as a psychologist or therapist). Please include disorders even if you did not need treatment for them or if you did not agree with the diagnosis”. Participants that endorsed current or previous diagnosis of a mental health problem or preferred not to answer were excluded. Further exclusion criteria were left-handedness, addiction or dependence on substances (not cigarettes or coffee) or behaviours (e.g. gambling), shift-work and non-white ethnic background (all indexed by self-report).

### Imaging acquisition parameters and processing

T_1_-weighted anatomical images for each participant were acquired on a single Siemens Skyra 3T scanner (Siemens, Erlangen, Germany) fitted with a 32-channel head coil according to previously reported procedures [30,31] with online documentation available here: http://biobank.ctsu.ox.ac.uk/crystal/docs/brain_mri.pdf. All image preprocessing and statistical analysis was performed using FSL v6.0.0 using recommended pipelines (see https://fsl.fmrib.ox.ac.uk/fsl/fslwiki/FSLVBM/UserGuide). Briefly, structural images were brain extracted to remove non-brain tissue and segmented into grey matter and white matter and cerebrospinal fluid. An unbiased study specific template was created from a random sample of participants (*N =* 1800) with an equal number of morning and evening types (900 participants from each type, equal numbers of males and females and similar in terms of age: MT, *M* = 55.15, SD = 7.74, ET, *M* = 54.43, SD = 7.29). To achieve this, segmented grey matter images from these participants were affine registered to the ICBM-152 template, concatenated and averaged, mirrored along the x direction and the two mirror images averaged to create an initial study specific affine template. In a second step, native space segmented grey matter images from each of these participants were then non-linearly registered to the initial affine template, concatenated, averaged, flipped (as above) and the resulting mirror images averaged to create a final symmetric, study-specific non-linear template in standard space. Native space segmented grey matter images from the entire cohort were then non-linearly registered to the study-specific template and modulation implemented to correct for local expansion or contraction due to the non-linear component of the spatial transformation. As the modulation Jacobian does not include the linear affine transformation this step effectively corrects for intracranial volume. Finally, an isotropic Gaussian smoothing kernel with sigma 3 was applied to the modulated images.

In the primary (hypothesis confirming) analysis a region of interest (ROI) was created using a sphere of 10 mm radius centred on the precuneus coordinates reported by Takeuchi *et al*., (2015) and used as a mask in the comparison between ET and MT. A General Linear Model (GLM) was applied to these data and tested for significance using non-parametric permutation tests (applying 5000 permutations). Control of the family-wise error rate was obtained using threshold-free cluster enhancement. Significance was defined at p ≤ .05. In a second exploratory analysis a whole-brain voxelwise General Linear Model (GLM) was applied to the data and tested for significance using non-parametric permutation tests (applying 5000 permutations). Control of the family-wise error rate was obtained using threshold-free cluster enhancement. Significance was defined at p ≤ .05. For both the ROI and the whole-brain exploratory analyses age was included as a covariate of no interest.

### Image quality control

Image quality control included visual inspection (RN) of initial image quality, following brain extraction and image registration. Participants with clear artifacts, poor quality brain extraction (substantial non-brain tissue not removed) or registration were excluded from subsequent analysis (please see Results for details).

## Results

Visual inspection of each individual’s brain extracted image revealed poor image quality/brain extraction or registration inaccuracy in 36 participants and these were excluded from subsequent analyses. A HTML file containing snapshots (images show each excluded participant’s anatomical image with the brain extracted image overlaid as red-outline) of the excluded participants is included as supplemental material (please see Supplemental materials below). The final sample (*N* = 3730) ages ranged from 40 to 70 years (*M* = 55.6), 51.7% of participants were female and morning types made up 74.5% of the sample. Evening types were younger than morning types (mean difference (years) 1.9, *p* < .001) but similar in terms of sleep duration (mean difference (hours) 0.01, *p* = .79. Please see Table 1 for a description of the full sample stratified by chronotype.

**Table 1:** MT = morning type, ET = evening type. Age in years (SD), sleep in hours.

Diurnal_preference	*N*	Age		Sleep		% Female

MT	2780	56.1	(7.21)	7.12	(1.01)	52
ET	950	54.2	(7.67)	7.11	(1.07)	49

**Figure 1:**
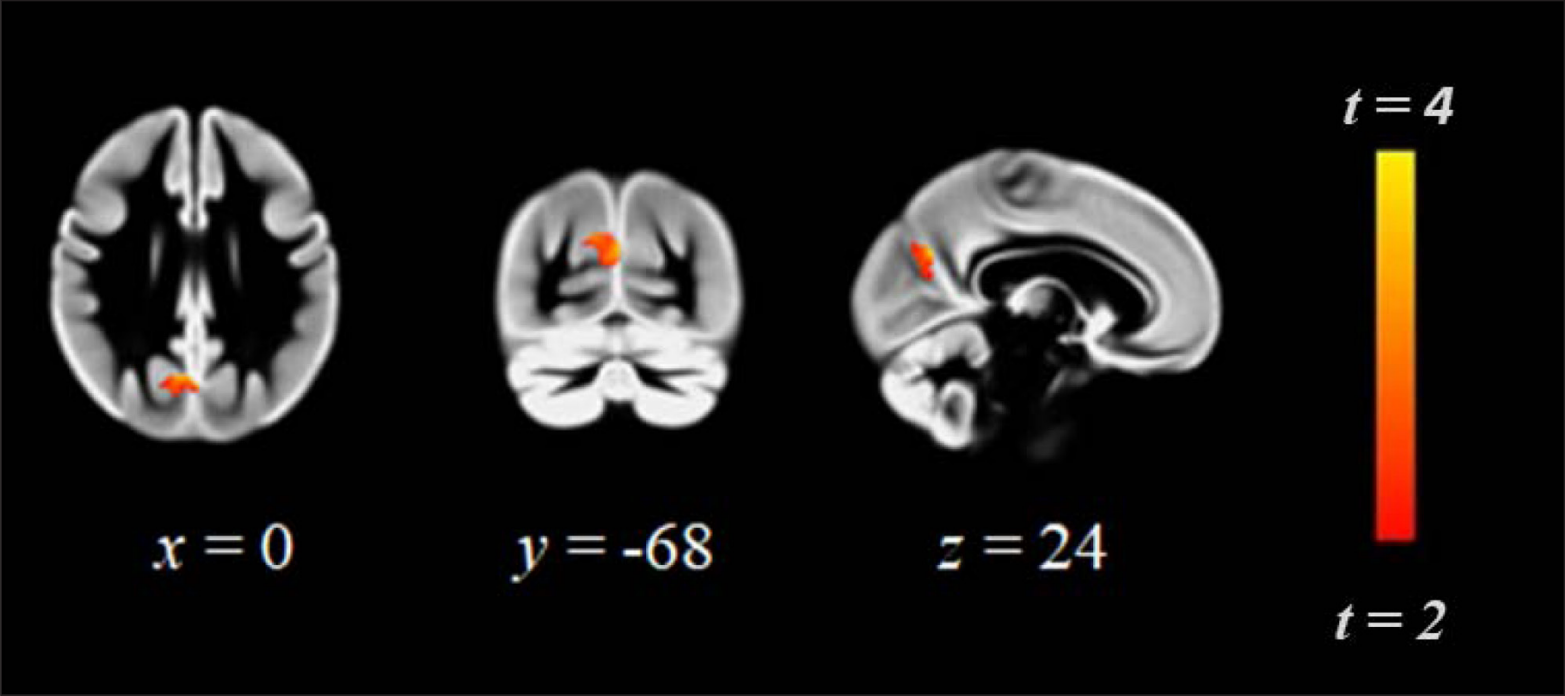
Region of greater precuneus grey matter volume in ET vs MT participants (for cluster details please see text). Displayed are sagittal, coronal and axial slices. Numerals refer to coordinates in MNI standard space. Images are presented in neurological space (left brain on right side).

**Figure 2:**
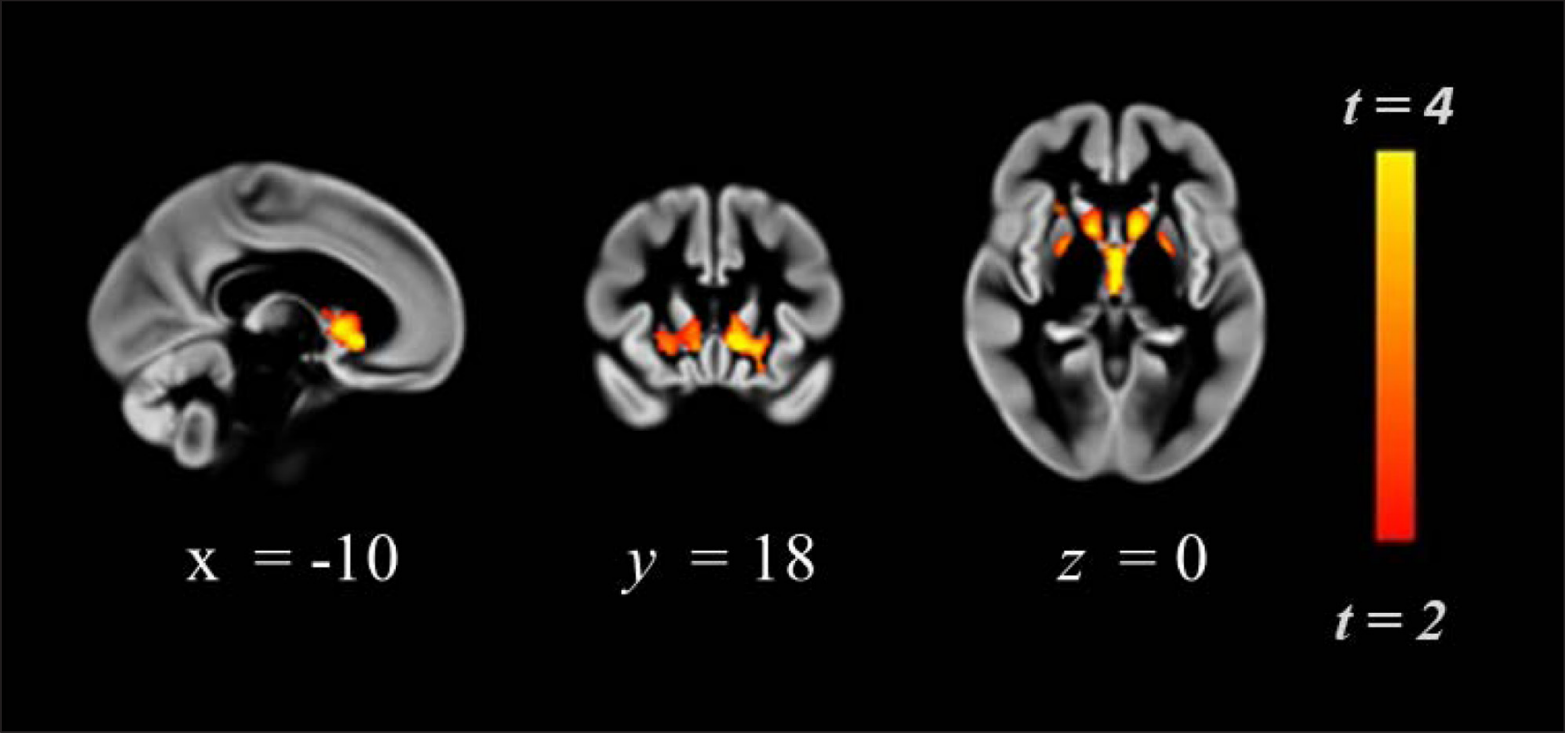
Region of greater thalamic/striatum GM volume in ET vs MT participants (for cluster details please see Table 2). Numerals as in figure 1.

Region of interest analysis demonstrated greater precuneal grey matter volume in ET as compared to MT: peak coordinates *x* = 0, *y* = –68, *z* = 24, cluster size = 184 voxels, maximum *t*-statistic = 2.74 (Figure 1). Whole brain VBM revealed reduced grey matter volume in key regions implicated in risk-taking and reward processing (bilaterally thalamus, nucleus accumbens, caudate, putamen and pallidum), left and right lateral orbitofrontal cortex and inferior frontal gyrus. Please see Table 2 and Figure 2.

**Table 2:** Whole brain voxelwise GLM. Data show region of peak activation, MNI coordinates, maximum t statistic and cluster size (voxels).

Region/s	Hemisphere	*X*	*Y*	*Z*	Maximum T statistic	Cluster size

Thalamus, NAcc, caudate, putamen, pallidum	R/L	0	–2	16	3.07	2240
Orbitofrontal cortex, frontal pole	R	32	38	–8	3.03	434
Inferior frontal gyrus	R	60	22	8	3.21	150
Orbitofrontal cortex, frontal pole	L	–46	42	–8	2.82	140
Orbitofrontal cortex, frontal pole	L	–40	30	–20	2.83	70

## Discussion

The current study aimed to explore the anatomical substrates of eveningness in a large population of older adults. Consistent with the study hypothesis definite eveningness was associated with greater grey matter volume in precuneus. Exploratory results (hypothesis-generating) revealed that eveningness was also associated with greater grey matter volume in bilateral thalamus and striatum and orbitofrontal cortex. There were no regions of greater grey matter volume in morning-types as compared to evening types.

Before discussing the specific results obtained here it is important to consider the strengths and limitations of this work. A key strength of the current work is the relatively large sample size. A key limitation was the use of a single question to assess diurnal preference. However, the question presented to Biobank participants (please see Methods) is similar to the final question of the full and reduced versions of the Morningness-Eveningness Questionnaire (“One hears about “morning types” and “evening types.” Which one of these types do you consider yourself to be?” [3,32]) which correlates strongly with total rMEQ score (*r* = .89, [33]). In addition, the UK Biobank cohort has been reported to be generally healthier than the general population [34]. The current findings may not, therefore, generalise to the entire population. Other factors, not measured here, may also have contributed to the current findings (e.g. diet, exercise) although current evidence suggests that ET tend to engage in less exercise and the frequency of eating-disorders and metabolic disorders is greater in this group [35]. How this might translate to greater grey matter volume in ET, which would be counterintuitive, if these effects are important, is unclear. Another important factor not measured here is social jetlag – the discrepancy between internal time and societal demands (e.g. work school etc). However, as the current sample included predominantly older adults the potential impact of social jetlag may be negated or reduced. Future studies that record dietary habits and indices of social jetlag are warranted.

Converging lines of evidence point to the precuneus as a key neural substrate for diurnal preference and chronotype. Recent resting-state FMRI studies have reported reduced precuneal functional connectivity in young adult late-type individuals as compared to morning-types [18,19] and structural MRI studies – again in younger adults – have demonstrated greater grey matter volume in the same region [28,29]. More generally, task-based FMRI studies indicate a role for the precuneus in a range of paradigms including social processing. For example, a recent meta-analysis utilising the Neurosynth database (a repository of over 14300 imaging studies: https://neurosynth.org) identified the precuneus as a key node in the human social brain [36]. Anatomically, reduced grey matter volume in this region has been associated with a number of pro-social traits including greater empathy [37,38] and agreeableness [39]. These data appear consistent with the findings of Randler who reported an association between morningness and agreeableness [40]. Morningness has also been associated with increased cooperativeness [41] and precuneus activation has been reported in FMRI tasks assaying cooperation and reciprocity [42]. Together, the current work and previous imaging studies [28,29] indicate the precuneus as an important anatomical substrate for diurnal preference in young and older adults and may underlie the proclivity for morning-types to engage in more pro-social behaviours.

Increasing evidence also suggests that eveningness is associated with indices of risk and reward [35] and here eveningness was associated with increased grey matter volume in a network of regions strongly implicated in risk and reward function. Previous work has demonstrated that young adult evening-types are reported to be more impulsive [43], show greater novelty and sensation-seeking [43,44,45], reduced harm-avoidance and persistence [44], greater general risk-taking across a number of domains including financial, ethical and recreational decision-making [46,47], substance use [48,49] increased coffee and stimulant use [50] and smoking [51]. More recently, Hwang *et al*. [52], observed an association between eveningness and greater impulsivity in a large sample (*N* = 1000) of community-dwelling adults (age range 20–77 years) thereby suggesting that the association between circadian typology and impulsivity is not restricted to youth and young adulthood.

Neuroimaging studies of risk and reward in relation to diurnal preference are limited. Hasler and colleagues [16] combined FMRI with a task designed to probe reward circuitry in thirty four male participants classified as morning- or evening-types based on the Composite Scale of Morningness. Compared to morning-types, evening-types showed reduced activation in a region of the medial prefrontal cortex during reward anticipation and greater striatal activation during win outcome. A pattern of activation the authors suggest is consistent with reduced regulatory control and elevated reward sensitivity in evening-type males. In a follow-up study Hasler *et al*. [15], reported that mPFC activation (in response to wins) mediated the effect of eveningness at baseline (age 20) on alcohol dependence at follow-up (age 22) thereby suggesting that altered reward-related brain function could underlie associations between eveningness and alcohol use problems [15].

To the author’s best knowledge this is the first study to report an association between grey-matter volume in the reward network and eveningness. As participants with current or previous psychiatric disorder and addiction or dependence on substances or behaviours were excluded the current findings are unlikely to reflect underlying psychopathology. Rather, they may reflect anatomical substrates of eveningness in healthy adults. The development imbalance model of impulsivity posits that adolescent impulsivity arises as a consequence of differential development of brain regions implicated in choice behaviour [53]. Approach behaviour is mediated by structures in the mesolimbic-dopamine reward system and is integrated with information from frontal regions that subserve impulse control to optimise the long-term goals of the individual. The slower development of frontal regions results in an imbalance between these competing systems favouring immediate gratification over long-term benefit. Consistent with this model Mackey and colleagues reported that greater impulsivity was associated with lower mediofrontal grey matter volume and greater grey matter volume in ventral striatum and anterior thalamus [54]. By contrast, Tschmegg *et al*., reported a positive association between grey matter volume in right caudate and impulsivity in a sample of 70 healthy adults [55] but no differences in mediofrontal regions. The authors suggest that hyper-activity in the mesolimbic-dopamine reward system accompanied by changes in fronto-striatal connectivity results in outlasting structural changes in striatum and thalamus that persists into adulthood in more impulsive individuals [55]. Eveningness has been associated with increased impulsivity in young [43] and older adults [52] and the current findings of increased grey matter volume in striatum and thalamus may reflect the neural substrate of increased trait impulsivity reported in evening-type individuals. Future studies that assess subjective and objective measures of impulsivity in conjunction with neuroimaging metrics of grey matter volume and functional connectivity are required.

In the current study eveningness was also associated with greater grey matter volume in bilateral orbitofrontal cortex. This is in contrast to the findings reported by Takeucki and colleagues [28] who observed an association between eveningness and lower grey matter volume in in orbitofrontal cortex and Rosenberg *et al*. [29], who observed no between-group differences in these regions. Lower lateral orbitofrontal grey matter volume has been observed in substance use disorder [56,57], substance dependence [58], a number of psychiatric disorders including depression [59,60] and internet-gaming disorder [61]. Eveningness has also been associated with substance use [48,49], depression [22,62,63,64] and with increased mobile-phone gaming [65]. The findings of Takeucki *et al*. [28], of lower grey matter volume in orbitofrontal cortex and the current work (greater grey matter volume) may be consistent with a risk-resilience model of eveningness. Young adult evening-types with lower orbitofrontal volume may be at increased risk for developing psychiatric disorder. By contrast, greater grey matter volume in older adults free from current or previous psychiatric disorder and addiction or dependence on substances or behaviours (the current study) may represent a resiliency factor. Future, suitably powered, prospective studies are required to fully test this hypothesis.

In summary, consistent with previous reports evening-type individuals displayed greater grey matter volume in precuneus. In addition, eveningness was associated with higher grey matter volume in bilateral nucleus accumbens, caudate, putamen and thalamus, orbitofrontal cortex. The current findings further support precuneus as an important neural substrate for diurnal preference and highlight grey matter differences for future study. It is possible that other factors not measured here (e.g. diet, social jetlag) impacted on the current findings. Nevertheless, well powered longitudinal studies are required to determine causality and the potential temporal effects of eveningness on brain structure.

## Additional Files

Open Science Framework (OSF): Diurnal preference and grey matter volume in a large population of older adults: Data from the UK Biobank – https://osf.io/8hzfb/

This project includes:

- Snapshot images of excluded participants
- Details of the study-specific template created for this report plus a compressed image file of the grey matter template
- Demographic details for all participants (in.csv; .omv and .rds formats).
